# Diabetic Neuropathy Is Associated With Lower Bone Mineral Density and Higher Fall Risk in Young Elderly Adults With Type 2 Diabetes

**DOI:** 10.1002/dmrr.70135

**Published:** 2026-02-08

**Authors:** Luca D'Onofrio, Rocco Amendolara, Antonio Siena, Alessandro Latino, Renata Risi, Angela Balena, Simona Zampetti, Davide Masi, Marianna Alfonsi, Barbara La Scaleia, Mikiko Watanabe, Myrka Zago, Francesco Lacquaniti, Ernesto Maddaloni, Raffaella Buzzetti

**Affiliations:** ^1^ Diabetology Unit AOU Policlinico Umberto I Rome Italy; ^2^ Department of Experimental Medicine Sapienza University Rome Italy; ^3^ Laboratory of Neuromotor Physiology IRCCS Santa Lucia Foundation Rome Italy; ^4^ Department of Systems Medicine and Centre of Space Bio‐medicine University of Rome Tor Vergata Rome Italy

**Keywords:** bone health, diabetic neuropathy, falls, insulin resistance, type 2 diabetes

## Abstract

**Background and Aim:**

Diabetic neuropathy (DN) is a recognised risk factor for fragility fractures. However, the mechanisms linking DN, bone health, and falling risk remain unclear. We aimed to assess bone health and risk of falls, with their contributing factors, in young elderly patients with type 2 diabetes (T2D) and mild‐to‐moderate DN.

**Methods:**

We enrolled 144 subjects with T2D, excluding those with severe DN (neuropathy disability score ‐NDS‐ ≥ 9) or fracture history. Clinical and biochemical data were collected, including surrogate markers of insulin resistance, such as the triglycerides/HDL (TG/HDL) ratio and triglycerides/glucose (TyG) index. Bone mineral density (BMD) and trabecular bone score (TBS) were evaluated using DXA scans. Falls were self‐recorded prospectively over 4 years using diaries.

**Results:**

Subjects with DN (27%) had higher BMI (*p* = 0.036), fasting blood glucose (*p* = 0.04), serum triglycerides (*p* = 0.016), TG/HDL ratio (*p* = 0.012) and TyG index (*p* = 0.003) compared with those without DN. After adjustment for gender, age, BMI, HbA1c, TyG index and TG/HDL ratio, subjects with DN showed significantly lower BMD at the femoral neck (0.702 [0.638–0.850] g/cm^2^ vs. 0.789 [0.717–0.860] g/cm^2^, *p* = 0.015) and total femur (0.890 [0.820–1.055] g/cm^2^ vs. 0.983 [0.889–1.076] g/cm^2^, *p* = 0.027). No differences were observed in spine BMD or TBS. However, TBS was negatively correlated with the TG/HDL ratio (*r* = −0.215, *p* = 0.013) and visceral adipose tissue (*r* = −0.310, *p* < 0.001). After 4 years of follow‐up, subjects with painful neuropathy at baseline had a higher rate of falls (*p* = 0.011).

**Conclusion:**

DN is associated with decreased BMD and increased risk of falls. Among factors associated with DN, insulin resistance was also associated with decreased bone quality.

## 1 Introduction

Type 2 diabetes (T2D) is one of the most prevalent chronic diseases around the world, affecting more than half a billion people worldwide, with a prevalence that has increased exponentially over the last years [1].

Alongside the well‐known micro‐ and macrovascular complications of diabetes, diabetic bone fragility is a newly established chronic complication of diabetes, characterised by impairment in bone quality and increased risk of bone fractures [2].

Factors underlying the development of diabetic osteopathy remain unclear, although several contributors have been hypothesised. In this regard, experimental and clinical studies suggest a bi‐directional relationship between classical diabetic vascular complications and increased bone fragility [3]. Among the chronic complications of diabetes, diabetic neuropathy could play a major role in fragility fractures by causing impaired postural stability and increasing the risk of falls [3].

Furthermore, hyperglycaemia exerts direct detrimental effects on osteoblastogenesis and osteoblast function, leading to reduced bone turnover [2, 4]. Contrasting data have been collected on the role of insulin resistance, as animal studies suggest an impairment in bone metabolism due to insulin resistance [5]; otherwise, clinical studies highlight contrasting and non‐conclusive results [2, 6].

Therefore, in this post hoc analysis, we aimed to investigate the impact of diabetic neuropathy and related risk factors on bone health and falls, the two main determinants of bone fractures. To this aim, we assessed the association between diabetic neuropathy and bone mass and quality in subjects with T2D, and we further longitudinally evaluated their risk of falls by the presence of diabetic neuropathy.

## 2 Materials and Methods

### 2.1 Study Design

This is a post hoc analysis of data collected within the SynErg (synergies anchoring to gravity) study, a cross‐sectional study designed to evaluate postural instability and falls in young elderly affected by T2D, as previously described [7]. Briefly, in the study, community‐dwelling individuals, aged between 65 and 75 years (young elderly), with a diagnosis of T2D based on the criteria of the American Diabetes Association [8], referring to the outpatient clinic of the Diabetology Unit of AOU (Azienda Ospedaliera Universitaria) Policlinico Umberto I, Sapienza University of Rome, were consecutively enrolled.

Subjects who could not perform the postural tests of the SynErg study, had foot ulcers or amputations, bone fractures, heart failure (NYHA 2–4), active cancer or a diagnosis of cancer within 5 years from enrolment, prior stroke, blindness, severe neuropathy (defined as a Neuropathy Disability Score—NDS ≥ 9), a history of severe hypoglycaemia, central nervous system disorders, dementia, dizziness, vertigo, spontaneous or positionally induced nystagmus, or vestibular migraine were excluded from enrolment.

### 2.2 Data Collection

Clinical (age, gender, disease duration, hypoglycaemic therapy, smoking status, blood pressure), biochemical (HbA1c, fasting blood glucose, lipid profile, triglycerides, creatinine) and anthropometric (weight, body mass index—BMI) data were collected for all participants. LDL cholesterol concentrations were estimated with the Friedewald formula: total cholesterol—[HDL + (triglycerides/5)] [9]. The estimated glomerular filtration rate (eGFR) was estimated according to the methods proposed by Levey and colleagues [10]. Further, information about treatment with anti‐diabetic agents (metformin, dipeptidyl peptidase‐4 inhibitors—DPP4‐i, glucagon‐like peptide‐1 receptor agonists—GLP1‐ra, sodium‐glucose co‐transporter inhibitors—SGLT2‐i, basal or rapid insulin) was also collected.

Dual‐energy X‐ray absorptiometry (DEXA—Hologic A Inc., Bedford, MA, USA, QDR 4500 W) was used to evaluate both BMD and trabecular bone score (TBS). After calibration of the DEXA scan according to standard procedures, subjects were placed in the lying position and, according to the manufacturer's instructions, a single experienced technician performed the examination. Bone mass density (BMD) assessment of the lumbar spine (L1–L4 anteroposterior), total femur and femoral neck (non‐dominant limb) was performed and BMD was recorded in terms of the absolute mineral content (in g/cm^2^) at various sites.

The integrated software TBS iNsight, version 2.1.2.0, was used for the site‐matched spine scans for the evaluation of TBS, a novel tool that estimated the microarchitecture and bone quality [11]. TBS values > 1.350 were considered normal, values below 1.350 indicated a reduced bone quality [11].

Whole‐body fat mass (fat mass, %) and abdominal visceral adipose tissue (VAT, g) were also evaluated with DEXA scans, QDR2000 Product Software, version 13.5.3 A (Hologic, Middlesex County, MA, USA).

Neuropathy disability score (NDS) was used to evaluate and stratify the presence of DN. In particular, pain sensitivity was assessed with pinprick perception (0 = normal, 1 = altered), thermal sensitivity was evaluated with temperature sensation (0 = normal, 1 = altered), vibratory sensitivity (0 = normal, 1 = altered), and the presence of Achilles reflexes (0 = normal, 1 = present with reinforcement, 2 = altered) was evaluated in both limbs. Hence, we could establish the absence of DN (≤ 2) and the presence of DN as mild (3–5), moderate (6–8) or severe (≥ 9) [12].

DN4 (Douleur Neuropathique 4) was used to identify neuropathic pain. It consists of 10 items, including 7 patient‐reported symptoms (such as burning, electric shocks, tingling, pins and needles, numbness, itching, and paraesthesia) and 3 clinical signs (hypoesthesia to touch and pinprick, and allodynia to light touch) assessed by the clinician. A score of four or more out of 10 was used to evaluate the presence of neuropathic pain [13].

The following surrogate markers of insulin resistance were assessed: the triglycerides glucose (TyG) index was evaluated using the formula proposed by Guerrero‐Romero and colleagues (Ln[fasting triglycerides (mg/dL) × fasting glucose (mg/dL)]/2) [14]; Triglycerides/HDL (TG/HDL) ratio was evaluated as previously published [15].

The enrolled subjects were followed for 4 years to assess the number of occurring falls, which were self‐recorded in a written diary and subsequently reviewed by the study investigators through in‐person visits or telephone calls. At the baseline visit, the subjects were instructed to recognise a fall according to the definition of World Health Organisation: ‘a fall is an event which results in a person coming to rest inadvertently on the ground or floor or other lower level’ [16] to avoid the reporting of near‐fall defined as a stumble or loss of balance leading to a potential fall, prevented only by recovery actions.

### 2.3 Statistical Analysis and Ethics

Data were expressed as frequencies, means and standard deviations or medians and interquartile ranges. The Shapiro‐Wilk test was used to assess the normal distribution of the variables. Student's *t*‐tests or the Mann‐Whitney, based on the normal or non‐normal distribution of the variables, were used to assess differences between the two groups. The frequencies of the variables were compared using the *χ* ^2^ test or Fisher's exact test. Pearson's or Spearman's coefficient was used to test for correlations between biochemical parameters and NDS score, TG/HDL ratio and TyG index. Finally, linear regression analysis (univariate and multivariate) was conducted to test the influence of clinical variables on neuropathy and bone parameters. In particular, gender, age, BMI, HbA1c, TyG index, TG/HDL ratio and treatment with GLP1‐ra were selected to evaluate the possible impact of gender and age, obesity, glucose metabolism and insulin resistance on the relationship between bone metabolism and neuropathy. In the linear regression model used, we added one variable at a time to evaluate how they affect the analysis and separately tested the TyG index and TG/HDL ratio to avoid collinearity.

The threshold value of statistical significance was reached for values of *p* ≤ 0.05. Statistical analysis was performed with IBM SPSS Statistics software (version 21). Graphs were created using Prism 9.0 for Windows (GraphPad Software, La Jolla, CA, USA).

This study was performed in accordance with the tenets of the Declaration of Helsinki and was approved by the Ethical Committee of AOU Policlinico Umberto 1 (prot. 111/20, *n*. 5635). Each participant enrolled in the study provided informed consent before starting the study procedures.

## 3 Results

This study population consisted of 144 subjects affected by T2D (*n* = 72% male) with a median [25th–75th percentile] age of 69 [66–72] years, disease duration of 8.5 [4.3–13] years, BMI of 28.3 [25.6–31.3] kg/m^2^ and HbA1c of 6.5 [6.0–7.2]%. Thirty‐nine (27%) had a NDS ≥ 3, allowing a diagnosis of DN.

Clinical features of subjects with and without DN are reported in Table 1. Subjects with DN showed higher BMI (29.7 [26.7–33.2] kg/m^2^ vs. 27.7 [25.5–30.8] kg/m^2^, *p* = 0.036), fasting blood glucose (127 [112–153] mg/dL vs. 119 [103–136] mg/dL, *p* = 0.04), serum triglycerides (136 [97–178] mg/dL vs. 105 [80–153] mg/dL, *p* = 0.016), TG/HDL ratio (3.3 [1.9–4.1] vs. 2.4 [1.5–3.5], *p* = 0.012) and TyG index (4.9 ± 0.3 vs. 4.7 ± 0.3, *p* = 0.003) compared to subjects without DN.

**TABLE 1:** Anthropometric, clinical and biochemical data in subjects with and without DN.

	No DN	Yes DN	*p* value*
	*n* = 105	*n* = 39	
Gender, male % (*n*)	73 (77)	69 (27)	0.625
Smoker, yes % (*n*)	12 (13)	10 (4)	0.726
Age, years	69 [66–72]	69 [66–71]	0.901
Disease duration, years	8 [4–12.5]	9 [5–13]	0.465
Weight, kg	82 ± 14	86 ± 17	0.234
BMI, kg/m^2^	27.7 [25.5–30.8]	29.7 [26.7–33.2]	**0.036**
Systolic blood pressure, mmHg	130 [120–135]	130 [120–140]	0.260
Diastolic blood pressure, mmHg	80 [70–80]	80 [70–80]	0.253
Fasting blood glucose, mg/dL	119 [103–136]	127 [112–153]	**0.040**
HbA1c, %	6.4 [6.0–7.1]	6.6 [6.0–7.5]	0.202
Total cholesterol, mg/dL	156 ± 38	159 ± 47	0.738
HDL cholesterol, mg/dL	47 [40–55]	46 [39–53]	0.455
LDL cholesterol, mg/dL	83 ± 22	84 ± 38	0.765
Triglycerides, mg/dL	105 [80–153]	136 [97–178]	**0.016**
Creatinine, mg/dL	0.9 [0.8–1.0]	0.9 [0.8–1.2]	0.863
eGFR	83.5 ± 22.4	81.2 ± 24.6	0.634
Total fat, %	26.0 [23.3–31.0]	26.5 [22.8–34.6]	0.436
VAT mass, g	809 ± 267	870 ± 251	0.220
Tg/HDL ratio	2.4 [1.5–3.5]	3.3 [1.9–4.1]	**0.012**
TyG index	4.7 ± 0.3	4.9 ± 0.3	**0.003**

^*^ *p*‐value in bold are for significant findings, *p* < 0.05.

No differences were reported in terms of gender, smoking habits, age, disease duration, weight, systolic blood pressure (PAS), diastolic blood pressure (PAD), HbA1c, lipid profile, creatinine, and eGFR. Anti‐diabetes treatment also did not differ between subjects with and without DN (Table S1). Total fat mass and VAT mass were similar between subjects with and without DN (Table 1).

**FIGURE 1:**
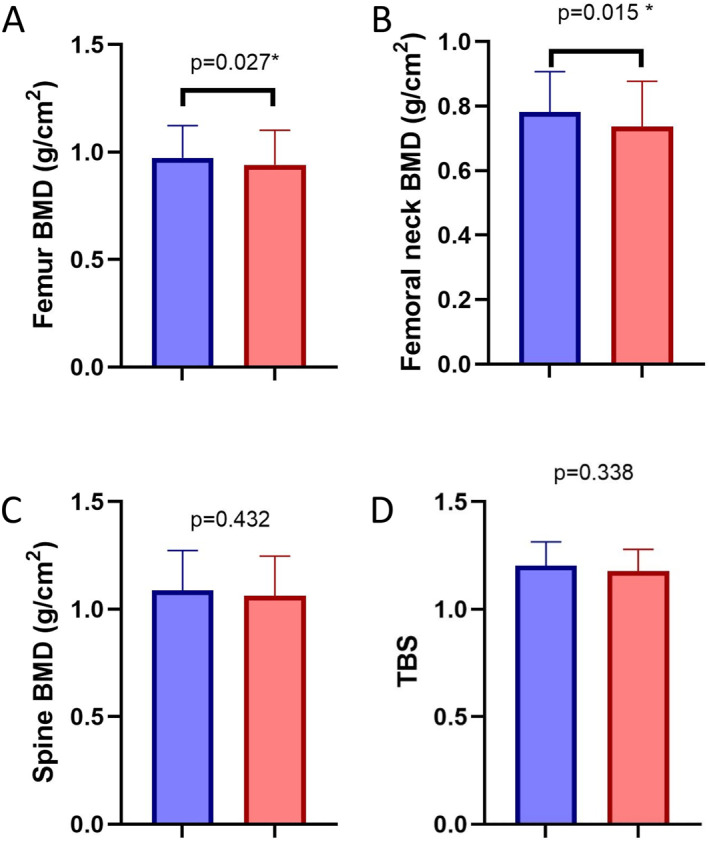
Differences in femur BMD (A), femoral neck BMD (B), spine BMD (C) and TBS (D) in subjects with (red) and without (blue) DN. * after adjustment for gender, age, BMI, HbA1c, TyG index and TG/HDL ratio.

After multivariate correction (gender, age, BMI, HbA1c, TyG index and TG/HDL ratio), DN was associated with reduced total femur BMD (0.890 [0.820–1.055] g/cm^2^ vs. 0.983 [0.889–1.076]g/cm^2^, *p* = 0.027, Figure 1A) and lower femoral neck BMD (0.702 [0.638–0.850] g/cm^2^ vs. 0.789 [0.717–0.860] g/cm^2^, *p* = 0.015, Figure 1B), compared to subjects without DN.

No significant differences were found in the total spine BMD (Figure 1C), as well as in the TBS values between subjects with and without DN (Figure 1D).

Subjects treated with GLP1‐ra showed higher values of total femur BMD compared with subjects not using GLP1‐ra (0.990 [0.898–1.094] vs.. 0.930 [0.830–1.058], *p* = 0.023). The association between DN and reduced total femur BMD remained significant when controlling for GLP1‐ra treatment in the multivariate model. Other anti‐diabetes drugs were not significantly associated with femur BMD.

**FIGURE 2:**
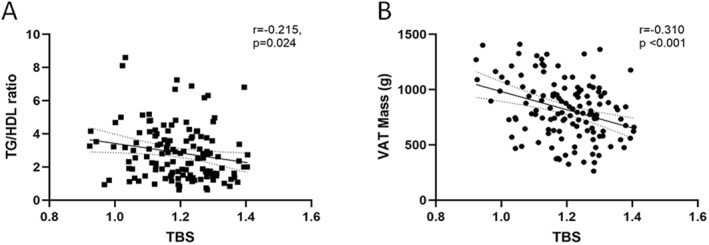
Correlation between TBS with TG/HDL ratio (A) and VAT mass (B).

An inverse relationship between the TG/HDL ratio and TBS was also found (*r* = −0.215, *p* = 0.024—Figure 2A). As expected, the TG/HDL ratio was directly related to BMI (*r* = 0.253, *p* = 0.002) and VAT (*r* = 0.240, *p* = 0.004).

Finally, an inverse correlation was found between VAT and TBS (*r* = −0.310, *p* < 0.001—Figure 2B).

### 3.1 Fall Risk

After four years of follow‐up, 140 subjects completed the falls diary, and data for only four subjects were not retrieved due to the patient's death. Hence, 24 subjects reported at least one fall, and 16 of them reported more than one fall.

Participants with painful neuropathy at baseline had a higher fall rate than those without (71.4% vs. 26.5%, *p* = 0.011). No differences were observed by diabetic neuropathy assessed with NDS at baseline (*p* = 0.438). Women experienced significantly more falls during the follow‐up period compared with men (28.6% vs. 23.5%, *p* = 0.030). No significant differences were found in subgroups by obesity status (*p* = 0.778). Patients who experienced falls also had a longer disease duration compared to those who did not fall (median 10 [5–17] vs. 8 [3–11] years, *p* 0.02).

The use of anti‐diabetes treatments (use of metformin, DPP4‐i, GLP1‐ra, SGLT2‐i, basal or rapid insulin) did not significantly differ between fallers and non‐fallers.

## 4 Discussion

In our study involving young‐elderly subjects with T2D, those with DN exhibited significantly reduced BMD at both the femoral neck and the total femur. Furthermore, the detrimental impact of insulin resistance on bone quality was supported by the negative association between surrogate markers of insulin resistance and VAT with TBS, a radiological marker of bone quality.

It is well‐established that diabetic osteopathy is a chronic complication of diabetes [2, 5]. Although both type 1 diabetes (T1D) and T2D are associated with an increased risk of fracture [17, 18], some studies have reported normal or even increased BMD in subjects with T2D despite impaired bone quality [19]. Several mechanisms have been postulated to explain the detrimental effect of diabetes on bone, including chronic hyperglycaemia and vascular complications. While an association between DN and bone fragility has been consistently shown in people with T1D [20, 21], findings in T2D remain heterogeneous. In this regard, a meta‐analysis published in 2021 did not confirm a clear effect of DN on bone density or turnover in diabetes. However, the same authors emphasised that the limited number of available studies prevented strong conclusions and highlighted the need for additional evidence [22]. In the following years, limited new data have been generated. Specifically, no association was found in Asian subjects between the presence of DN and bone turnover markers [23].

Differently, an analysis of the ‘Fenofibrate Intervention and Event Lowering in Diabetes’ (FIELD) trial revealed that DN was independently associated with fragility fractures in women [24]. More recently, a post hoc analysis of the Exenatide Study of Cardiovascular Event Lowering (EXSCEL) similarly reported an increased risk of fractures in people with T2D affected by DN [25]. A retrospective study in Chinese subjects found a higher prevalence of DN among subjects with osteoporosis, although the association was lost after correction for confounders [26].

Finally, nerve conduction velocity was positively associated with BD in men, but not in women, with T2D [27].

Considering this conflicting evidence, we aimed to evaluate BMD and TBS in young‐elderly people with and without mild‐to‐moderate DN. Our results revealed a significant reduction in bone mass among those with DN regardless of gender, BMI, and HbA1c levels, highlighting the need for further confirmatory research.

We also found that painful neuropathy was correlated with an increased risk of falls. This suggests that different types of neuropathies may have different impacts on fall risk. Painful neuropathy may increase fall risk due to multifactorial contributors, including impaired balance, muscle weakness, reduced postural control and psychological consequences such as fear of falling or loss of confidence in daily activities. This highlights the importance of differentiating painful neuropathy from other neuropathic conditions when assessing fall risk, and planning targeted interventions.

It is well known the impact of hypoglycaemic treatment on bone metabolism [28], and in our cohort we confirm a positive impact of GLP1‐ra on total femoral BMD; however, this finding has no effect on the association between DN and femoral BMD.

Our findings also confirm that women and subjects with longer diabetes duration experienced more falls, as previously reported in other studies [29, 30].

Amongst the well‐known risk factors for neuropathy, we also confirm the detrimental role of triglycerides, as previously demonstrated in people with diabetes and severe hypertriglyceridemia [31, 32].

The TG/HDL ratio, a commonly used clinical marker of insulin resistance, was found to correlate with TBS. suggesting that higher levels of insulin resistance may negatively impact bone quality in T2D. Indeed, insulin resistance, through an impairment in insulin signalling in osteoblasts, could reduce osteoblast function, affecting bone metabolism [33]. Moreover, in a small sample of people, TBS was negatively associated with insulin resistance and visceral adipose tissue, supporting our findings [34]. Further, the negative association between VAT, one of the key contributors to insulin resistance [35], and TBS strengthens these findings. The relevance of TBS in subjects with obesity and diabetes, especially in T2D [5], is well known as a marker of bone impairment.

Taking into consideration all this evidence, on the other side, it is also important to consider the hypothesis that bone mass and bone quality could be affected differently by DN and insulin resistance, and longitudinal studies are needed to better elucidate this hypothesis.

This study has several limitations but some important strengths. The cross‐sectional design prevents causal inference regarding the impact of DN bone impairment. Generalisability is limited due to the specific characteristics of the SynErg cohort, which included young elderly people aged 65–75 years, without severe neuropathy, foot ulceration, amputation, previously fragility fractures, and with the ability to perform postural assessments. This may introduce selection bias. Moreover, the post hoc analysis design highlights the need for dedicated studies assessing the longitudinal effects of DN on both BMD and TBS. Nonetheless, the narrow age range and the exclusion of advanced neuropathy reduce confounding by age and severe diabetic foot complications. The extensive phenotyping of participants, including TBS and body composition by DEXA, further strengthens our findings.

In conclusion, this post hoc analysis demonstrated an association between DN and reduced bone mass in young elderly subjects with T2D and identified painful neuropathy as a contributor to fall. Insulin resistance significantly differed between people with and without DN and was associated with markers of bone quality in T2D. Prospective and mechanistic studies are needed to clarify whether DN and diabetic osteopathy share common insulin resistance‐mediated pathological pathways and to determine whether interventions targeting insulin resistance may improve both DN and bone health outcomes.

## Author Contributions

L.D.O. designed the study, enrolled subjects, data analysis and wrote the manuscript. A.S. and M.A. enrolment of subjects and data collection. A.L., R.R., A.B., D.M., B.L.S. and M.W. performed DEXA examination, neuropathy evaluation and data collection. R.A. and S.Z. performed laboratory analysis, reviewed/edited the manuscript. M.Z. and F.L. revised the manuscript, E.M. data analysis and wrote the manuscript. R.B. contributed to discussion and revised the manuscript.

## Conflicts of Interest

The authors declare no conflicts of interest.

## Supporting information


**Table S1:** Hypoglycemic treatment in subjects with and without DN.

## Data Availability

The data that support the findings of this study are available upon request from the corresponding author. The data are not publicly available due to privacy or ethical restrictions.
