# Supplementary material for: Diabetic Neuropathy Is Associated With Lower Bone Mineral Density and Higher Fall Risk in Young Elderly Adults With Type 2 Diabetes
Source: Diabetes Metab Res Rev. 2026 Feb 8;42(2):e70135. doi: 10.1002/dmrr.70135 (PMC12883200; doi:10.1002/dmrr.70135)
Supplement: Supplementary file 1 — Table S1: Hypoglycemic treatment in subjects with and without DN. [file DMRR-42-e70135-s001.docx]

**Supplementary Table 1. Hypoglycemic treatment in subjects with and without DN.**

|  | no DN  n = 105 | yes DN  n = 39 | p |
| --- | --- | --- | --- |
| Metformin, % (n) | 88.6 (93) | 97.4 (38) | 0.099 |
| DPP4-i, % (n) | 4.8 (5) | 10.3 (4) | 0.226 |
| GLP1RA, % (n) | 46.7 (49) | 41.0 (16) | 0.546 |
| SGLT2-i, % (n) | 23.8 (25) | 38.5 (15) | 0.081 |
| Basal insulin, % (n) | 19.0 (20) | 30.8 (12) | 0.133 |
| Rapid insulin, % (n) | 3.8 (4) | 5.1 (2) | 0.725 |

DPP4-i: dipeptidyl peptidase 4; GLP1RA: glucagon-like peptide-1 receptor agonist; SGLT2i: sodium-glucose co-transporter 2 inhibitor
